# Effects of dihydrocapsiate on adaptive and diet-induced thermogenesis with a high protein very low calorie diet: a randomized control trial

**DOI:** 10.1186/1743-7075-7-78

**Published:** 2010-10-06

**Authors:** TszYing Amy Lee, Zhaoping Li, Alona Zerlin, David Heber

**Affiliations:** 1 Center for Human Nutrition, David Geffen School of Medicine, University of California, Los Angeles, CA 90095, USA

## Abstract

**Background:**

Dihydrocapsiate (DCT) is a natural safe food ingredient which is structurally related to capsaicin from chili pepper and is found in the non-pungent pepper strain, CH-19 Sweet. It has been shown to elicit the thermogenic effects of capsaicin but without its gastrointestinal side effects.

**Methods:**

The present study was designed to examine the effects of DCT on both adaptive thermogenesis as the result of caloric restriction with a high protein very low calorie diet (VLCD) and to determine whether DCT would increase post-prandial energy expenditure (PPEE) in response to a 400 kcal/60 g protein liquid test meal. Thirty-three subjects completed an outpatient very low calorie diet (800 kcal/day providing 120 g/day protein) over 4 weeks and were randomly assigned to receive either DCT capsules three times per day (3 mg or 9 mg) or placebo. At baseline and 4 weeks, fasting basal metabolic rate and PPEE were measured in a metabolic hood and fat free mass (FFM) determined using displacement plethysmography (BOD POD).

**Results:**

PPEE normalized to FFM was increased significantly in subjects receiving 9 mg/day DCT by comparison to placebo (p < 0.05), but decreases in resting metabolic rate were not affected. Respiratory quotient (RQ) increased by 0.04 in the placebo group (p < 0.05) at end of the 4 weeks, but did not change in groups receiving DCT.

**Conclusions:**

These data provide evidence for postprandial increases in thermogenesis and fat oxidation secondary to administration of dihydrocapsiate.

**Trial registration:**

clinicaltrial.govNCT01142687

## Introduction

Resting energy expenditure (REE) decreases in response to caloric restriction [1-4]. In the obese, this decline in REE results in a decreasing rate of weight loss during periods of low calorie dieting. There have been attempts to inhibit the adaptive decrease in energy expenditure that occurs through aerobic exercise [5-7], but a previous study from our group [8] demonstrated that the institution of aerobic exercise did not affect the adaptive decrease in thermogenesis which is believed to be mediated by a change in sympathetic nervous system (SNS) activity.

Capsaicin from chili pepper is known to stimulate thermogenesis through a central nervous mechanism, but at doses required to observe this metabolic effect intolerable gastrointestinal side effects occur. Studies suggest that capsinoids such as dihydrocapsiate (DCT) found in the non-pungent CH-19 sweet pepper share the positive metabolic characteristics of capsaicin without inducing gastrointestinal side effects [9-13]. DCT has a hot taste threshold estimated at approximately 1,000 times that of capsaicin, but both capsaicin and DCT stimulate TRPV1 receptors in the gut which bring about activation of the SNS, which can increase lipogenesis and thermogenesis [10]. Extracts of CH-19 sweet have been shown to increase body temperature, oxygen consumption, sympathetic nervous system activation, and to lead to weight loss in two studies [11,14].

The present study was designed to examine the effects of DCT on both adaptive thermogenesis as the result of caloric restriction with a high protein very low calorie diet (VLCD) and to determine whether DCT would increase post-prandial energy expenditure (PPEE) in response to a 400 kcal/60 g protein liquid test meal.

## Subjects and methods

### Subjects

The study was approved by the Institutional Review Board of the David Geffen School of Medicine at the University of California, Los Angeles. Healthy men and postmenopausal women with body mass index (BMI) between 27 and 35 kg/m^2 ^were recruited through internet advertisement and campus flyers. Subjects were excluded for conditions of type 2 diabetes or glucose intolerance, significant weight loss within 3 months, history of bariatric surgery, a history of alcohol or cigarette use, eating disorders, depression treated with medications and chronic diseases other than controlled hypertension and hypercholesterolemia. The study was registered at clinicaltrial.gov, NCT01142687.

### Study Protocol

Each subject gave informed consent prior to beginning the study. At the screening visit medical history, physical exam, electrocardiogram and fasting blood tests were performed. All eligible subjects were then started on the lead-in phase when all were instructed to consume maintenance calories and record food intake for 7 days to assure that they maintain constant weight within 1 kg. Subjects were then randomized double-blinded into placebo, 3 mg or 9 mg DCT groups for 4 weeks. Vital signs, anthropomorphic assessments, safety labs and adverse events were assessed weekly. The blood samples were sent to the UCLA Clinical Laboratory for analysis. Body composition and indirect calorimetric measurements are performed at day 0 and 28.

### Supplements

The dihydrocapsiate capsules of 1 mg and placebo were obtained from Ajinomoto Co (Ajinomoto Co., Inc. Japan). Dihydrocapsiate was synthesized enzymatically with vanillyl alcohol and 8-methylnonanoic acid. Following the esterification, filtration, extraction and evaporation are conducted. Refined rapeseed oil was used to dilute dihydrocapsiate to adjust the concentration. 200 mg of concentration adjusted dihydrocapsiate was packed in soft capsules, followed by drying. The placebo capsules to be used for the control group were prepared in the same manner as the dihydrocapsiate capsules.

The investigators and subjects were blinded to the identity of the capsules. Each subject was instructed to consume capsules containing either DCT or placebo three times per day within 30 minutes before breakfast, lunch and dinner in a manner designed to provide 0, 3, or 9 mg of DCT per day.

### Very Low Calorie Diet (VLCD)

The VLCD plan consisted of eight servings per day of a very low calorie protein shake providing 100 kilocalories and 15 grams of high quality protein (Pro-Cal, R-Kane Products). Forty miliequivalent of K-Dur (potassium chloride tablet) were provided to all the subjects at the start of the meal plan and the dose was adjusted according to the weekly safety labs to maintain the potassium level within the normal range. Subjects consumed four additional servings on testing day one and day 28. Subjects were counseled by the registered dietician and physician for product compliance and possible side effects from the capsules.

### Body composition

Body weight was measured using a calibrated electronic scale and body composition was measured using an air displacement body plethysmograph (BOD POD, Life Measurements Instruments). Subject wore a swimsuit, biking shorts and a swim cap during the measurement.

### Indirect calorimetric measurement

At day 0 and 28, subjects arrived at the facility in a fasting state. Basal metabolic rate (BMR) was obtained by measuring oxygen consumption and carbon dioxide production over 20 minutes using a Vmax hood (Cardinal Systems) while subjects remained recumbent at room temperature. Subject consumed a high protein test liquid meal over twenty minutes providing 400 kilocalories and 60 grams of protein. Oxygen consumption and carbon dioxide production was measured hourly over the next four hours for postprandial energy expenditure (PPEE). PPEE were adjusted by fat free mass (FFM).

### Statistical analysis

Statistical analyses were carried out using SAS version 8.0.02 and a p value of 0.05 specified for statistical significance. For the evaluation of body weight and fat mass, the changes from baseline among groups were evaluated using 1-factor ANOVA. For the evaluation of changes in PPEE/FFM and RQ, the differences in mean change at each time point for different doses were evaluated using a mixed model with the change as a response variable and with dose, time point and dose-time point interaction as fixed effects. For evaluation of changes in BMR, the differences of mean changes with different doses were evaluated by using a mixed model with changes as response variables, and with doses as fixed effects.

## Results

### Baseline characteristics

Out of 58 subjects screened, 46 subjects met the eligibility criteria and were randomized into the study. They were randomly assigned to receive three times per day placebo (n = 15), DCT 3 mg (n = 15), or 9 mg (n = 16). The numbers of dropped subjects in the three groups above were 4, 3, and 6 respectively. One subject was withdrawn because of elevated blood pressure which was an exclusion criteria not noted at the time of entry. The remaining subjects were dropped due to lack of dietary compliance with the VLCD program. None of subjects withdrew as the result of any adverse reaction to the study supplements. Thirty-three subjects completed the study.

The demographic data are shown in Table 1 and there were no statistical differences among the groups. The BMI of subjects ranged from 26.9 to 38 kg/m^2^. Twenty-eight (61%) subjects were White, 7 (15%) were Hispanic, 8 (17%) were Black, 2 (4%) were Asian, and 1(2%) was Native American.

**Table 1:** Baseline characteristics

		DCT	
	Placebo	3 mg	9 mg
Subject number	15	15	16
Age (y)	49.6 ± 2.3	51.3 ± 2.6	53.9 ± 2.5
Females	5	6	9
Weight (kg)	92.8 ± 3.4	87.8 ± 4.8	89.9 ± 3.4
BMI (kg/m^2^)	30.9 ± 0.9	30.4 ± 0.7	31.3 ± 0.7
Resting metabolic rate (kcal/d)	1730.9 ± 71.4	1658.1 ± 101.0	1649.9 ± 100.0

### Change of body weight and body composition

**Figure 1:**
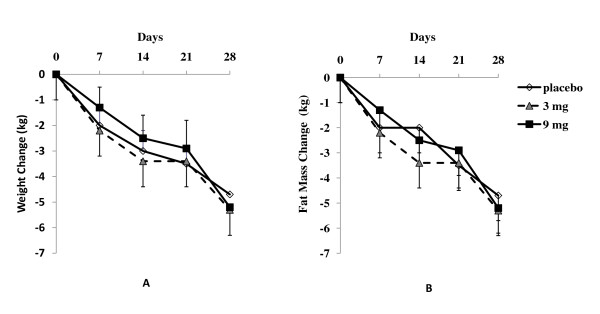
**Change of body weight (A) and fat mass (B)**. Fat mass was determined by BODPOD. Values are shown mean and SE.

All subjects lost significant amounts of weight over the four weeks. The average weight loss was 4.7 ± 0.6, 5.3 ± 0.7, and 5.2 ± 1.1 kg respectively for placebo, 3 mg and 9 mg DCT groups. As shown in Figure 1A, there were no significant differences in weight loss over the four weeks of the study among the three groups. The results of fat mass measured by BOD POD are shown in Figure 1B. No difference in overall fat mass lose was observed between the groups.

### Basal metabolic rate

**Figure 2:**
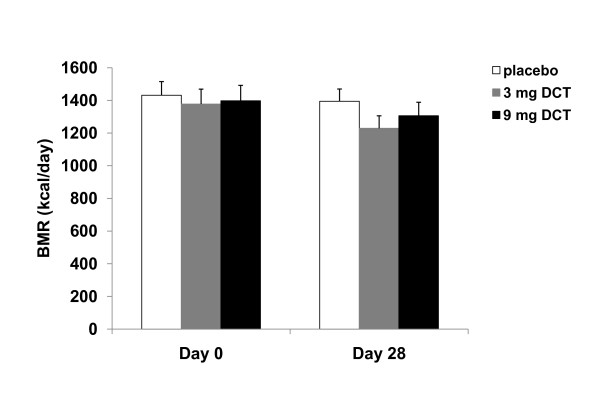
**Average basal metabolic rate (BMR) at day 0 to day 28**.

**Figure 3:**
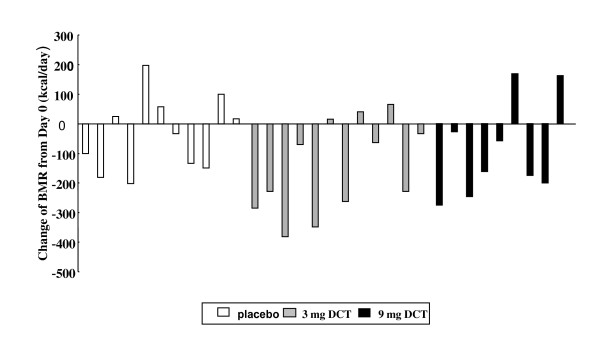
**Individual change of basal metabolic rate (BMR) from day 0 to day 28**.

As shown in Figure 2, there was not any significant change in BMR after 4 weeks of very low calorie diet in the three groups. Variation with BMR in response to the VLCD was observed in all three groups. (Figure 3). DCT did not change the adaptive decrease in metabolic rate secondary to VLCD.

### Postprandial energy expenditure

**Figure 4:**
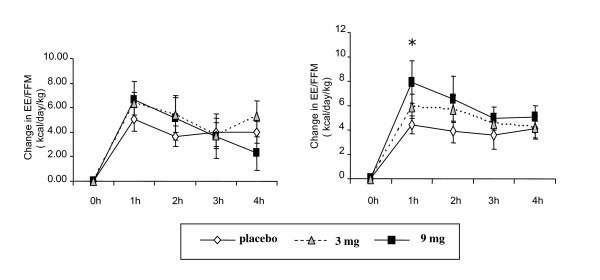
**Post-prandial energy expenditure (PPEE) at day 0 (A) and day 28 (B)**. Change (from 0 h) in PPEE adjusted by fat free mass (PPEE/FFM). FFM was determined by BODPOD. Values are shown mean and SE. * represents a significant difference (P < 0.05) when compared vs. placebo.

**Figure 5:**
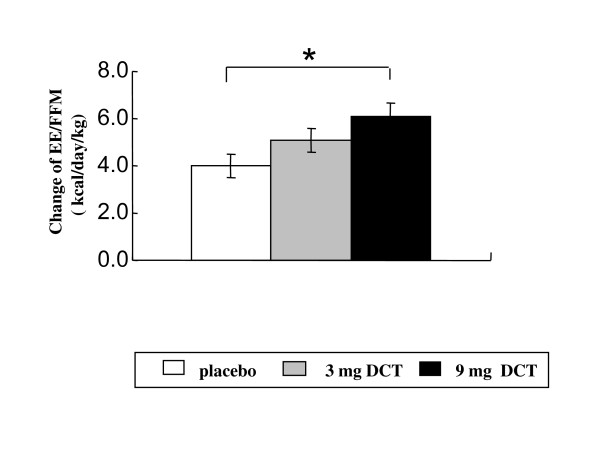
**Changes of average of energy expenditure adjusted by fat free mass (PPEE/FFM) from 1 h to 4 h during diet induced thermogenesis at Day 28**. FFM was determined by BODPOD. Values are shown mean and SE. * represents a significant difference (P < 0.05) when compared vs. placebo.

All subjects increased PPEE/FFM at day 0 in response to the test meal. There was no significant difference among the groups. Compared with subjects in the placebo group, there was an increase in PPEE/FFM at 1 hour at day 28 for subjects in the 3 mg and 9 mg but only the change in the 9 mg was statistically significant (p < 0.05) (Figure 4). When the average PPEE/FFM change over the four hours after the test meal was compared among the groups, there was a dose-dependent increase in PPEE/FFM at doses of DCT 3 and 9 mg at day 28 and the average PPEE change in 9 mg DCT group was significantly higher than the placebo group (Figure 5).

### Respiratory quotient

**Figure 6:**
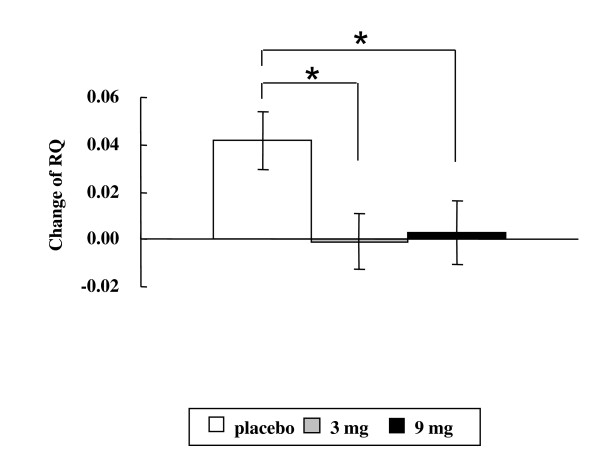
**Changes of average of respiratory quotient (RQ) from 1 h to 4 h during diet induced thermogenesis at Day 28**. Values are shown mean and SE. * represents a significant difference (P < 0.05) when compared vs. placebo.

The average respiratory quotient was 0.75 ± 0.02, 0.80 ± 0.02 and 0.76 ± 0.01 for placebo, DCT 3 mg and 9 mg respectively. At day 28, there was an increase of in average post-prandial RQ of 0.04 for subjects receiving the placebo while the DCT groups did not change. This increase in RQ was significant in comparison with the DCT groups consistent with decreased fat oxidation in the placebo group (p < 0.05) (Figure 6)

## Discussion

The capsinoids are found in all tested cultivars of the *Capsicum *genus of plants which includes chili peppers [15,16]. The capsinoids include capsiate, dihydrocapsiate, and nordihydrocapsiate which are structural analogs of the capsaicin, dihydrocapsaicin, and nordihydrocapsaicin found in hot chili peppers. Capsaicin results in a hot sensation in the oral cavity, but the capsinoids are not perceived as hot because they are hydrolyzed as they cross the oral mucosa. Capsinoids including dihydrocapsiate stimulate thermogenesis in animals, and activate the neuronal TRPV1 receptors on vagal afferent nerves in the intestine leading to increased sympathetic nervous system activity with uncoupling of oxidative phosphorylation leading to heat production [10,17].

In this small double-blinded, randomized, placebo controlled trial, we found that the consumption of a nonpungent capsiate supplement by overweight and obese subjects significantly increased post-prandial energy expenditure when combined with a high protein, very low calorie diet on an outpatient basis. The increase is estimated to be equivalent to an increase in daily energy expendtiture of approximately 100 kcal for a 100 kg individual. Hill et al using data from national surveys have estimated that changing energy balance favorably by 100 kilocalories per day could prevent the secular trends in weight gain observed in the U.S. population [18].

Total daily energy expenditure is composed of basal metabolic rate, postprandial thermogenesis, and physical activity thermogenesis. An adaptive reduction in EE in response to energy restriction can be observed in any component of total EE, traditionally divided into resting and non-resting EEs (including physical activity and non-exercise activity EEs), and thermic effect of food [19]. However, these changes are usually observed in resting EE in individuals undergoing dietary restriction for longer periods of time. In this study there was individual variation on the adaptive thermogensis in our study subjects at 4 weeks. There were no significant changes observed at the group level in any of our study groups after 4 weeks of VLCD and DCT supplementation. Inoue et al. previously reported that a significant increase of oxygen consumption was detected in the subjects with BMI over 25 in resting status when they ingested capsinoids for 4 weeks [12]. In this context, a marked reduction of body weight by VLCD in this study may have more impact on resting energy expenditure than that of capsinoids.

Subjects in all three groups lost significant amounts of body weight and fat mass but there were no significant differences observed in weight or fat loss among the groups. The severe restriction of calorie intake in the VLCD may have overshadowed any potential effects of capsinoids. While there were prior investigations establishing the effects of capsaicin and capsiates on satiety, the VLCD did not allow assessment of food intake as a practical measure of effects on satiety [20,21].

In this clinical trial, the impact of capsiate supplementation was tested on an outpatient basis where compliance with diet is always problematic and undoubtedly influenced the observations made here. This small metabolic study was not designed to assess long-term effects on weight and body composition.

It has been reported in several small clinical trials that capsinoids suppress body fat accumulation [11,13,22]. Josse et al has recently reported that 10 mg of capsinoids resulted in a shift on substrate utilization toward lipid [23]. Using indirect calorimetry, we observed that DCT supplementation blunted the increase in post-prandial respiratory quotient at day 28 seen in the placebo group suggesting that DCT increased fat oxidation. Further studies are needed to assess the impact of the predicted differences in energy expenditure related to DCT supplementation on weight loss and weight maintenance in free-living populations.

## Competing interests

The authors declare that they have no competing interests.

## Authors' contributions

ZL and DH designed the research and wrote the paper; TL, ZL and AZ conducted the research. All authors read and approved the final manuscript.
